# Establishment of a chicken intestinal organoid culture system to assess deoxynivalenol-induced damage of the intestinal barrier function

**DOI:** 10.1186/s40104-023-00976-4

**Published:** 2024-02-18

**Authors:** Tae Hong Kang, Sang In Lee

**Affiliations:** 1 Department of Animal Science and Biotechnology, Kyungpook National University, Sangju, Gyeong-Sangbuk-Do 37224 Republic of Korea; 2 Research Institute for Innovative Animal Science, Kyungpook National University, 37224, Sangju, Gyeong-Sangbuk-Do Republic of Korea

**Keywords:** Barrier function, Deoxynivalenol, Organoids

## Abstract

**Background:**

Deoxynivalenol (DON) is a mycotoxin that has received recognition worldwide because of its ability to cause growth delay, nutrient malabsorption, weight loss, emesis, and a reduction of feed intake in livestock. Since DON-contaminated feedstuff is absorbed in the gastrointestinal tract, we used chicken organoids to assess the DON-induced dysfunction of the small intestine.

**Results:**

We established a culture system using chicken organoids and characterized the organoids at passages 1 and 10. We confirmed the mRNA expression levels of various cell markers in the organoids, such as *KI67*, leucine-rich repeat containing G protein-coupled receptor 5 (*Lgr5*), mucin 2 (*MUC2*), chromogranin A (*CHGA*), cytokeratin 19 (*CK19*), lysozyme (*LYZ*), and microtubule-associated doublecortin-like kinase 1 (*DCLK1*), and compared the results to those of the small intestine. Our results showed that the organoids displayed functional similarities in permeability compared to the small intestine. DON damaged the tight junctions of the organoids, which resulted in increased permeability.

**Conclusions:**

Our organoid culture displayed topological, genetic, and functional similarities with the small intestine cells. Based on these similarities, we confirmed that DON causes small intestine dysfunction. Chicken organoids offer a practical model for the research of harmful substances.

**Supplementary Information:**

The online version contains supplementary material available at 10.1186/s40104-023-00976-4.

## Background

The global consumption of poultry is increasing because of its many advantages over other meats, including its lower price, product consistency, high protein levels, low fat content, and adaptability [1]. The feed that is used in poultry farming is an important aspect of poultry production, and it accounts for a high proportion of the overall livestock production cost [2]. A major effect of global warming and the associated increased CO_2_ concentrations in the atmosphere on agricultural productivity and the production of livestock feed is the increased generation of mycotoxins, which are toxic compounds that are produced by fungi. Mycotoxin-contaminated feed reduces agricultural productivity and product quality [3].

Deoxynivalenol (DON) is a mycotoxin that is produced by the *Fusarium* species and is most abundant in feedstuffs derived from grains, such as wheat, rye, barley, oats, and corn [4]. DON is resistant to processing, milling, and heating, which increases the risk that livestock will be exposed to DON-contaminated feedstuffs [5]. DON is rapidly absorbed in the gastrointestinal tract after consumption of contaminated feed, which then spreads to the other organs via plasma and accumulates in the intestines over time [6]. DON causes the dysfunction of protein and nucleic acid biosynthesis by binding to the ribosomes, which disrupts the cellular proliferation, apoptosis, and differentiation. The intestine and liver are high protein turnover organs and are the main targets of DON cytotoxicity [5, 7]. DON also damages the epithelial integrity and reduces the repair and replacement rates of epithelial cells through its negative effects on the intestinal barrier [8].

The gastrointestinal tract plays an important role in product performance, immunity, metabolism, health, digestion, and nutrient absorption [9]. The epithelium of the small intestine consists of a single layer of cells that are securely joined by junctions, which include tight and adherens junctions [10]. The intestinal epithelium’s homeostasis is maintained by stem cells that are found in the crypts, which produce daughter cells and are known as transit amplification cells. The transit amplification cells have the ability to differentiate into various cell types, such as goblet cells, Paneth cells, tuft cells, and enteroendocrine cells [11]. These cell types make up the intestinal epithelium and play an important role in nutrient absorption and as the intestinal barrier against the invasion of external environmental factors, including toxins, microorganisms, and pathogens [12].

Organoids mimic the functionality and organization of organ tissues and have become an invaluable tool for the in vitro study of biological processes, development, and diseases [13–16]. In the field of livestock biotechnology, organoid-based systems have been used for disease modeling, the determination of host pathogens interactions, and nutritional research to improve the productivity of livestock [17–19]. Organoids can be created from adult stem cells, pluripotent stem cells, embryonic stem cells, and intestinal stem cells (ISCs) when cultured under the appropriate conditions. These cells undergo self-renewal, morphogenesis, self-organization, and differentiation within the crypt villus domain [20, 21]. Small intestinal organoids display topological, genetic, and functional similarities to the small intestine; however, organoid research on DON-induced dysfunction remains lacking.

This study aimed to develop an organoid culture system to evaluate the effect of DON on nutrient absorption, barrier function, and organoid characterization. Further, we considered whether this culture system mimics the small intestine epithelium function to assess its adequacy for use as a replacement for studies on toxic substances in animals. This research will improve the understanding of DON’s effect on the small intestine barrier function and will improve overall chicken productivity.

## Methods

### Isolation of small intestine stem cells, intestinal organoid culture, and treatment

Three small intestine fragments from the duodenum, jejunum, and ileum were obtained from 20-day-old chicken embryos (Hy-Line Brown). The tissues were washed with a phosphate-buffered saline solution (PBS, Thermo Fisher Scientific, Wilmington, DE, USA) that contained 1% penicillin/streptomycin (Thermo Fisher Scientific). The washed fragments were sliced into pieces, collected in 15-mL conical tubes, and centrifuged at 244 × *g*. The supernatant was then removed, and the collected (jejunum, duodenum, and ileum) pellets were incubated with a cell dissociation solution (StemCell Technologies, Vancouver, BC, Canada) for 1 h in a shaker to release the stem cells. The pellets were then disrupted using a pipette and washed with Dulbecco’s Modified Eagle Medium (DMEM; Thermo Fisher Scientific). The pellets were filtered using cell strainers (diameter 100-μm and 70-μm), the supernatant was discarded, and the stem cells (100–150 stem cells) were seeded in an intestinal human organoid medium (StemCell Technologies) and Matrigel (Corning, USA) at a 1:1 ratio in a 24-well culture plate. The organoid cells were incubated in a CO_2_ incubator for 20 min, and 1 mL intestinal human organoid medium was added. The organoids were then incubated at 37 °C in a 5% CO_2_ incubator. To assessment by DON-induced barrier function damage in treatment group, organoid cells were incubated at concentration DON 2 μg/mL for 24 h.

### Passage and cryopreservation of chicken intestinal organoids

The organoid cells were incubated for 3–4 d until reaching one passage. The medium was then removed, and the cells were incubated with a cell dissociation solution for 1 min. The organoid cells were then transferred to a 15-mL conical tube and incubated for 10 min, the supernatant was removed after the suspension was centrifuged at 244 × *g*, and the organoid cells were washed using DMEM and centrifuged again at 244 × *g* for 5 min. The pellets were then seeded in an intestinal human organoid medium and Matrigel at a 1:1 ratio in a 24-well culture plate. The organoid cells were incubated in a CO_2_ incubator for 15 min, and then 1 mL intestinal human organoid medium was added. The medium was changed once every 3–4 d. To cryopreserve the organoids, the media supernatant was discarded and the cells were incubated with a cell dissociation solution for 1 min. The Matrigel matrix was disrupted by pipetting, and the supernatant was transferred to a 15-mL conical tube and incubated on a shaker at 50 r/min for 10 min. The supernatant was centrifuged at 244 × *g* for 5 min, and the collected organoid pellet was washed with DMEM, and centrifuged again at 244 × *g* for 5 min. The organoid cell pellet was suspended in a 90% human intestinal organoid medium with 10% dimethyl sulfoxide for cryopreservation (Sigma-Aldrich, Saint Louis, MO, USA). The organoid cells were stored frozen overnight and transferred to a liquid nitrogen tank for long-term preservation.

### Immunofluorescent staining of chicken intestinal organoids

The organoid medium was then discarded, and the organoid cells were fixed using 4% paraformaldehyde for 15 min at room temperature. The organoids were permeabilized in a buffer containing 0.5% Triton X-100 (Sigma-Aldrich) for 30 min at room temperature. The organoid cells were blocked using a buffer (1× PBS, 5% normal goat serum, 0.3% Triton X-100) for 1 h. The appropriate antibodies, including F-actin (Cat. No. AB6016, Millipore, Massachusetts, USA), villin (Cat. No. SC-58897, Santa Cruz, Texas, USA), E-cadherin (Cat. No. 610181, BD biosciences, New Jersey, USA), and chromogranin A (Cat. No. BS-0539R, Bioss, Massachusetts, USA), were used in a 1:200 antibody dilution solution (1× PBS, 1% BSA, 0.3% Triton X-100) and incubated overnight at 4 °C. The organoid cells were washed three times with PBS, and appropriate secondary antibodies (647; Cat. No. ab150115, 594; Cat. No. ab150080, Thermo Fisher Scientific) were added at a 1:500 ratio. The organoid cells were then stained with DAPI (Vector Laboratories, Burlingame, CA, USA), and the stained samples were seeded and mounted on glass slides. Representative photographs were obtained using a confocal microscope (LSM 900 with Airyscan 2, Oberkochen, Germany).

### Quantitative real-time PCR and Western blot assay

The total RNA was extracted using the AccuPreP Universal RNA Extraction kit (BioNEER, Daejeon, Republic of Korea). RNA (1 μg) was used to generate the first-strand cDNA. The primers for the target genes were designed using Primer 3 (http://frodo.wi.mit.edu) for qPCR. The following conditions were used for the qRT-PCR: 95 °C for 3 min; followed by 40 cycles at 95 °C for 15 s; 56–58 °C for 15 s; and 72 °C for 15 s. The results were normalized to glyceraldehyde-3-phosphate dehydrogenase (*GAPDH*), and the expression levels of target genes were calculated using the 2^−ΔΔCt^ method. The primer sequences of the genes are presented in Table 1.

**Table 1:** List of primers

Genes	Description	Accession No.	Seqeunce (5'→3')
*LGR5*	Leucine rich repeat containing G protein-coupled receptor 5	XM_425441	GTG ACC AGT TGC CTA ATC TCC
			CGG AAT GTG TCT GCT TTG AT
*MUC2*	Mucin 2	XM_040701656	TGG TCT CTG TGG GGA TTA CA
			CCG AGT TTC ATC AGG GTC TT
*CK19*	Cytokeratin 19	NM_205009	CGT TGG AAG AAG CAA ACT CA
			CGA TAG TGG CAG CAA GGA T
*CHGA*	Chromagranin A	XM_421330	ATT TCT ATC CTT CGC CAC CA
			TGC CAC ATC TCT TTG CTT GT
*DCLK1*	Doublecortin like kinase 1	NM_001257257	AAG CCT ACG GAA ACA GAG GA
			TAT TGA TGC TGG AAC CTG GA
*LYZ*	Lysozyme	NM_205281	ACC CAG GCT ACA AAC CGT AA
			CAG TTC ACG CTC GCT GTT AT
*SLC7A5*	Solute carrier familiy 7 member 5	NM_001030579.3	GGCTGTGGACTTTGGGAATC
			AGAAGCCTCGGGTGAATCAT
*GLUT1*	Glucose transperter 1	NM_205209.2	CTGTTGCTGGGCTGTCTAAC
			TCTCCGGCACCTTGAAGTAG
*GAPDH*	Glyceraldehyde-3-phosphate dehydrogenase	NM_204305	CCC CAA TGT CTC TGT TGT TG
			GTG GAG GAA TGG CTG TCA C

Proteins for Western blotting were extracted using a lysis buffer that contained a protease inhibitor cocktail (Cat. No. P3100-001, DAWINBIO, Gyeonggi-Do, Republic of Korea). The protein concentration was calculated using the A-pierce BCA Protein Assay kit (Thermo Fisher Scientific). After the protein samples were electrophoresed in a 6% polyacrylamide gel for 1 h at 100 V, they were transferred to a membrane for 90 min at 100 V. The membrane was then washed three times with Tris-buffered saline containing 0.1% Tween 20 (TBST) and blocked by 5% skim milk. The membrane was incubated over night with the primary antibody ZO-1 (Beta-actin, Santa Cruz Biotechnology, Dallas, TX, USA) and washed three times using TBST. The secondary antibody was used to treat the membrane for 1 h, and the membrane was visualized using an Enhanced Chemiluminescence (ECL) reagent. The images of the protein bands were obtained using the ChemiDoc imaging system.

### Apical-out organoid construction of chicken intestinal organoids

The medium was discarded, DMEM was added, and the Matrigel was disrupted by pipetting. The organoids were then centrifuged at 244 × *g* for 5 min and washed with PBS. The centrifugation step was repeated at 244 × *g* for 5 min, and the supernatant was discarded. Then 5 mmol/L cold EDTA/PBS was used to treat the organoid cells, which were then incubated on a shaker at 4 °C for 1 h. Thereafter, the organoid cells were centrifuged at 244 × *g* for 5 min, and the supernatant was discarded. The resulting pellets were washed with DMEM, suspended in an intestinal human organoid medium, and seeded into a low-attachment 24-well plate.

### Epithelial barrier permeability of chicken intestinal organoids

The function of the intestinal epithelial barrier was assessed using fluorescein isothiocyanate (FITC)-dextran (4 and 40 kDa; Sigma-Aldrich). The organoid cells were incubated for 3–4 d, and the medium was cleared. FITC-dextran was diluted in phenol red free DMEM. The organoid cells were treated with 25 ng/μL FITC-dextran for 30 or 60 min at room temperature. The organoid permeability was then observed under a fluorescence microscope (Korealabtech, Gyeonggi-Do, Republic of Korea).

### Fatty acid absorption of chicken intestinal organoids

The organoids were transferred to 15-mL conical tubes and centrifuged at 244 × *g* for 5 min. The supernatant was removed, and the cells were washed with non-phenol red DMEM and centrifuged for another 5 min at 244 × *g*. The supernatant was removed, and C1-BODIPY-C12 (Invitrogen, Wilmington, DE, USA) and fatty acid-free BSA were combined to a final concentration of 5 µmol/L, which was then added to the organoids. The organoids were then incubated at 37 °C in a CO_2_ incubator for 30 min. These fluorescent samples were seeded and mounted onto glass slides. Representative photographs were obtained with a confocal microscope (LSM 900 with Airyscan 2).

### Amino acid absorption of chicken intestinal organoids

The medium was discarded, DMEM without phenol red was added, the Matrigel was disrupted by pipetting, and the organoids were centrifuged at 244 × *g* for 5 min. The organoids were transferred to 15-mL conical tubes and washed twice with prewarmed Hanks balanced salt solution (HBSS, Sigma-Aldrich). The organoids were then incubated at 37 °C in a CO_2_ incubator for 5 min. The supernatant was removed, and the organoids were treated with a prewarmed bisphenol-A (BPA) uptake solution (a mixture of HBSS, BPA solution, and BPA dilution buffer) and then incubated at 37 °C in a CO_2_ incubator for 5 min. The supernatant was removed and washed three times with a prewarmed HBSS. The supernatant was removed, and the organoids were then treated with a prewarmed working solution (a mixture of HBSS and probe solution) (Cat. No. UP04, Dojindo, Kumamoto-ken, Japan) and incubated at 37 °C in a CO_2_ incubator for 5 min. The organoid cells were then stained with SYTO™ Deep Red Nucleic Acid Stain reagent (Thermo Fisher Scientific), and representative photographs were taken using a confocal microscope (LSM 900 with Airyscan 2).

### Glucose absorption of chicken intestinal organoid

The medium was discarded, DMEM without phenol red was added, the Matrigel was disrupted by pipetting, and the organoids were centrifuged at 244 × *g* for 5 min. The organoids were transferred to 15-mL conical tubes, washed using a glucose and serum-free medium, and incubated at 37 °C in a CO_2_ incubator for 15 min. The supernatant was then removed, and a prewarmed probe solution (a mixture of serum-free medium and glucose uptake probe) (Cat. No. UP02, Dojindo) was added to the organoids and incubated at 37 °C in a CO_2_ incubator for 15 min. The supernatant was removed, and the organoids were washed once with the kit WI solution and incubated at room temperature for 5 min. Representative photographs were taken using a confocal microscope (LSM 900 with Airyscan 2).

### Statistical analysis

Data analysis was performed using an SAS *t*-test to determine the significant differences between the treatment and control groups. The error bars indicate the standard error from analyses performed in triplicate. The significant differences compared to the control group are indicated using the following symbols: ^*^P < 0.05; ^**^P < 0.01; and ^***^P < 0.001. The differences were assessed using Duncan multiple-range tests.

## Results

### Stable culture of chicken intestinal organoids

**Fig. 1:**
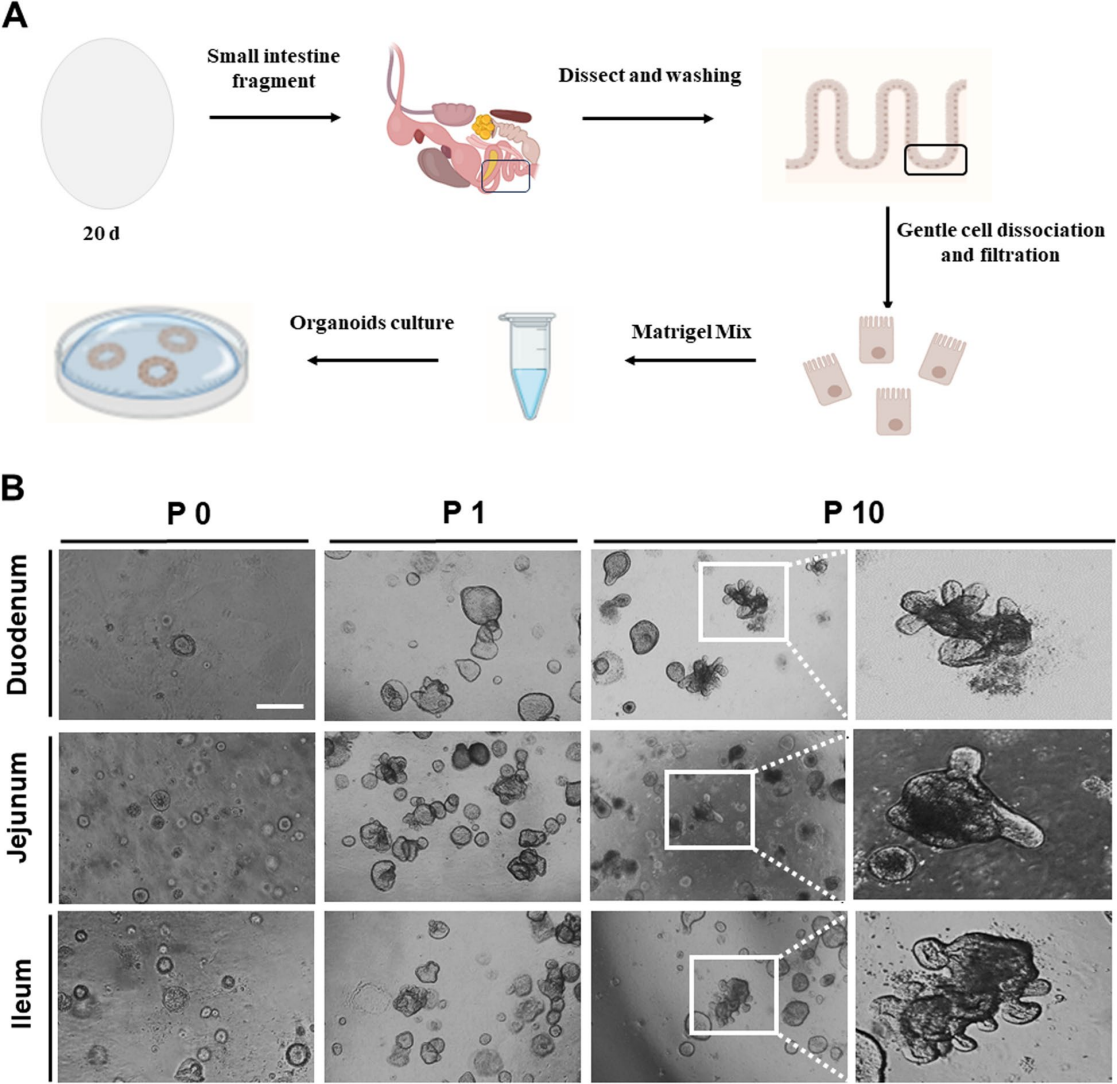
Development of chicken intestinal organoids. **A** Schematic illustration of the experimental procedure for the chicken organoid culture. **B** Development of the chicken organoids from passage 0 to passage 10. The organoid images were obtained using an optical microscope. Scale bar = 20 μm

The organoid cell culture procedures for the isolation of the intestinal stem cells in the chicken. Organoids are presented in Fig. 1A. The various organoid shapes, including spheroids (round-shaped), budding (spheroids with extension), mature villi, and crypt-like structures, were found from passage (P)0 to P10. At P0 (d 3), the organoids were mostly spheroids. As time progressed, the organoids tended to have a budding shape (P1, d 8), with mature villi and crypt-like structures that formed at P10 (d 39; Fig. 1B). These data indicate that the chicken organoids were successfully developed.

### Characterization of chicken intestinal organoid

**Fig. 2:**
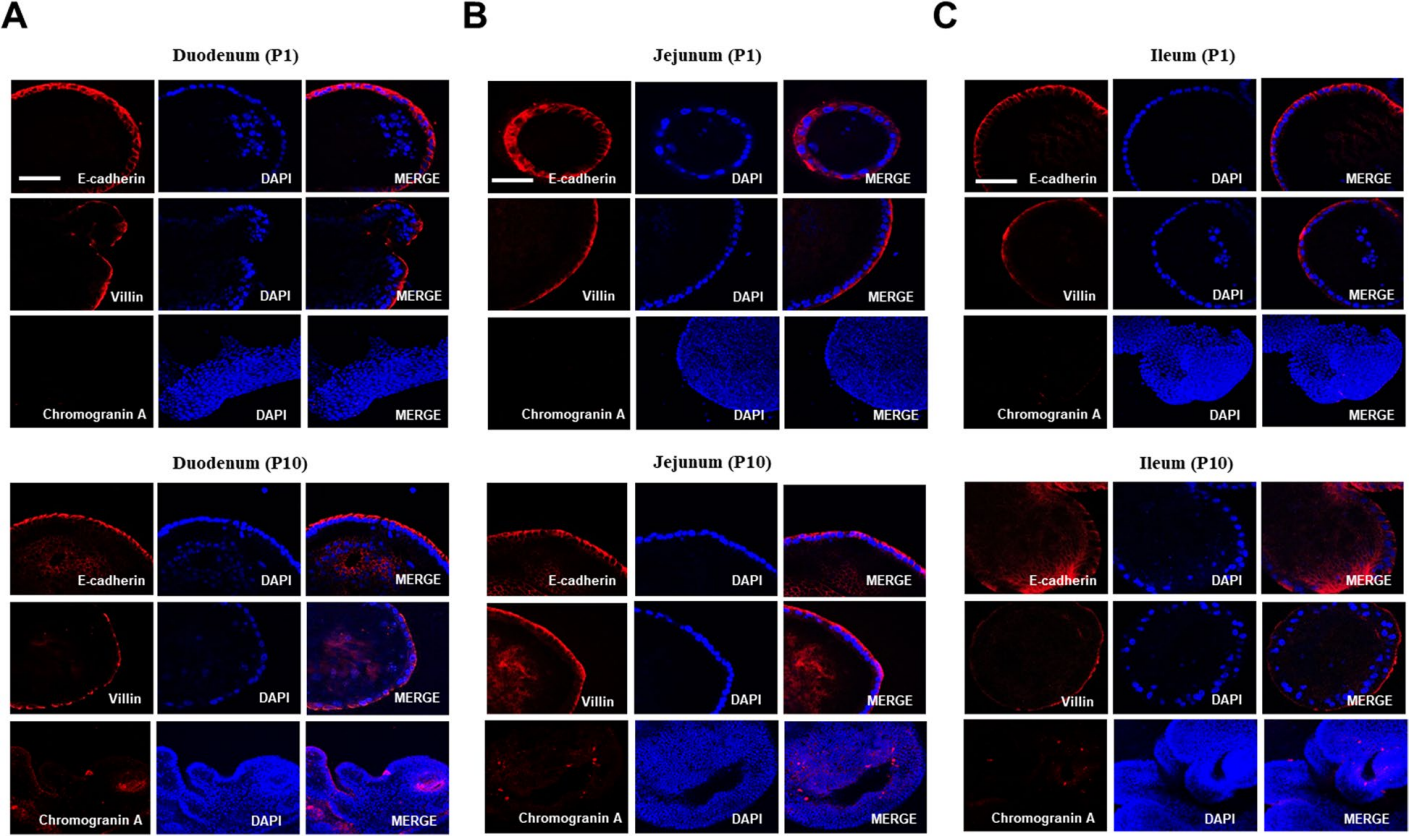
Characterization of chicken intestinal organoids. **A** Immunostaining of E-cadherin, villin, and CHGA of the chicken duodenum organoids at passage 1 and passage 10. **B** Chicken jejunum organoids were immunostained for E-cadherin, villin, and CHGA at passage 1 and passage 10. **C** Immunostaining of E-cadherin, villin, and CHGA of chicken ileum organoids at passage 1 and passage 10. Organoid immunostaining images were obtained under a confocal microscope. Nuclei were stained with 4′,6-diamidino-2-phenylindole (DAPI; blue). Scale bar = 20 μm

To characterize the cell diversity of the small intestine epithelium, we investigated the expression of various cell type markers, such as *CHGA* for enteroendocrine, E-cadherin for intestinal adherent junctions, and villin for the tissue-specific actin-binding proteins. E-cadherin and villin showed no differences between P1 and P10, and the expression of *CHGA* was detected at P10 compared to P1 (Fig. 2A–C). These data suggest that the developed organoids are genetically similar to the chicken small intestine because of the mature villus and the formation of crypt-like domains.

### Genetic similarity of chicken intestinal organoids

**Fig. 3:**
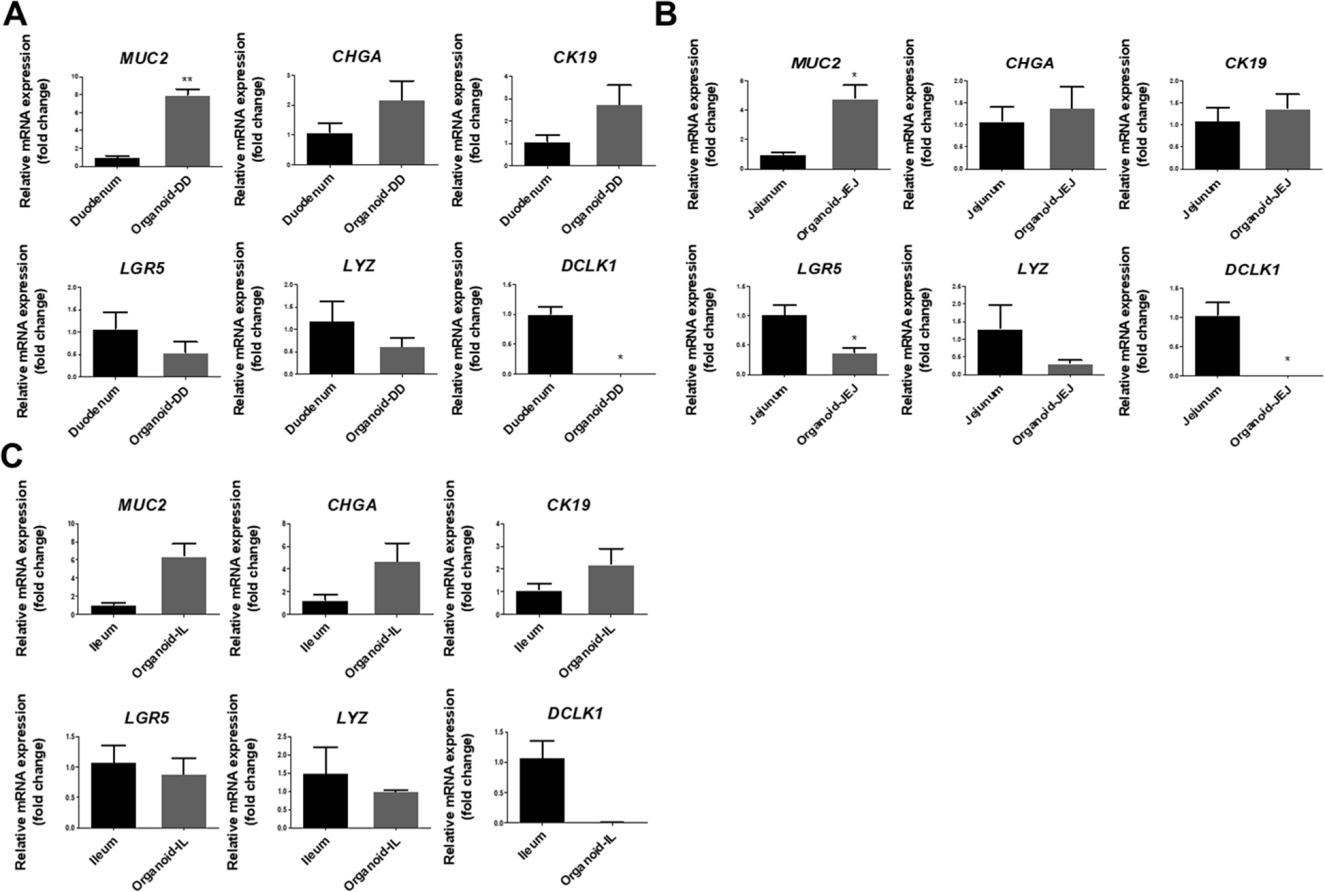
Genetic properties of chicken intestinal organoids. **A** Expression of the mRNA levels of *MUC2*, *CHGA*, *CK19*, *LGR5*, *LYZ*, and *DCLK1* in the chicken duodenum organoids compared to the actual chicken small intestine. **B** Expression of the mRNA levels of *MUC2*, *CHGA*, *CK19*, *LGR5*, *LYZ*, and *DCLK1* in the chicken jejunum organoids compared to the actual chicken small intestine. **C** The mRNA expression levels of *MUC2*, *CHGA*, *CK19*, *LGR5*, *LYZ*, and *DCLK1* in the chicken ileum organoids compared to the actual chicken small intestine. Error bars indicate the standard error (SE) of the analysis performed in triplicate. ^*^P < 0.05; ^**^P < 0.01; ^***^P < 0.001

We performed qRT-PCR to investigate the genetic properties of the chicken small intestinal organoids. The *MUC2* mRNA expression levels were significantly increased in the chicken organoids of the duodenum and jejunum compared to the actual chicken duodenum and jejunum. There was no difference in the *MUC2* mRNA expression levels of the ileum. The mRNA expression levels of *CHGA*, *CK19,* and *LYZ* were not significantly different in the organoids of the duodenum, jejunum, and ileum. Whereas *LGR5* was decreased in organoid jejunum compared to actual chicken jejunum (Fig. 3A–C). The duodenum and jejunum of the organoids had significantly decreased mRNA *DCLK* expression levels compared to the actual chicken duodenum and jejunum. However, the ileum of the organoids had no difference compared to the actual chicken ileum.

### Apical-out organoid formation for the assessment of small intestinal barrier function and deoxynivalenol-induced dysfunction

**Fig. 4:**
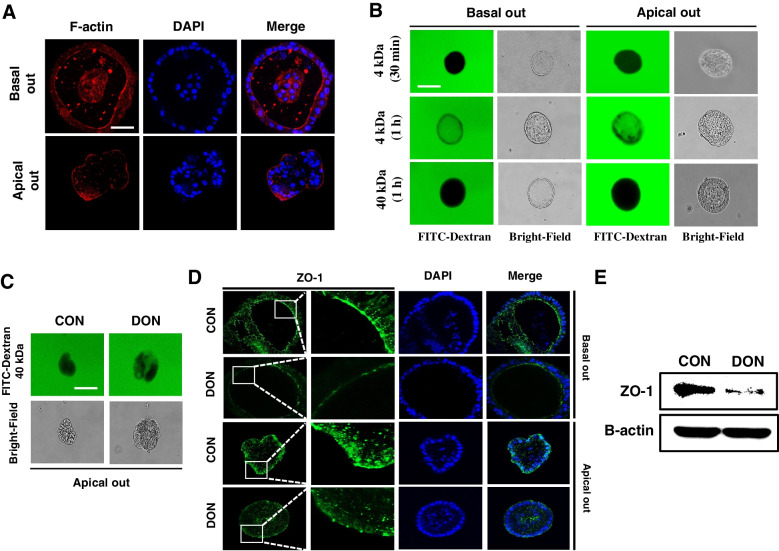
Paracellular permeability of chicken intestinal organoids and the small intestine dysfunction induced by deoxynivalenol. **A** Immunostaining of the apical-out organoids for the small intestine function assessment. **B** Paracellular permeability of the intestinal epithelial layer in the chicken organoids. **C** Dysfunction of the chicken intestinal epithelium barrier through deoxynivalenol-induced damage. **D** The expression of ZO-1 in the chicken organoids treated with 2 µg/mL deoxynivalenol compared with the untreated group. Nuclei were stained with 4′,6-diamidino-2-phenylindole (DAPI; blue). Scale bar = 20 μm. **E** The protein expression of ZO-1 when treated with 2 µg/mL deoxynivalenol compared with the untreated group

We obtained apical-out organoids that were verified using immunohistochemistry to investigate the epithelial barrier function and nutrient absorption in the small intestinal organoids. The filamentous actin (F-actin) expression sites were generally confirmed to identify the structural differences of the basal-out and apical-out organoids (Fig. 4A). In addition, we confirmed the paracellular permeability to determine whether the intestinal epithelial barrier function of the organoids was present using FITC-dextran (4 and 40 kDa). The 4 kDa FITC-dextran did not enter the lumen through the paracellular pathway, and the 40 kDa FITC did not enter the lumen (Fig. 4B). However, the 4 kDa FITC-dextran penetrated into the organoid lumen when the organoid was incubated with FITC-dextran for 1 h. These data indicate that the chicken organoid has a robust intestinal epithelial barrier, and the long-term treatment with FITC-dextran could be entered into lumen due to time effect. We evaluated the permeability assay and zonula occludin 1 (ZO-1) expression after the organoids were treated with DON to assess whether DON disrupted the intestinal barrier function. When treated with DON, more 40 kDa FITC-dextran entered the organoid lumen compared to the untreated organoids (Fig. 4C). In addition, the expression of ZO-1 was decreased (Fig. 4D and E). These results suggest a functional similarity between the constructed chicken organoids and the actual chicken small intestine, and DON caused epithelium barrier function disruption by reducing the expression of ZO-1.

### Assessment of nutrient uptake in chicken intestinal organoids

**Fig. 5:**
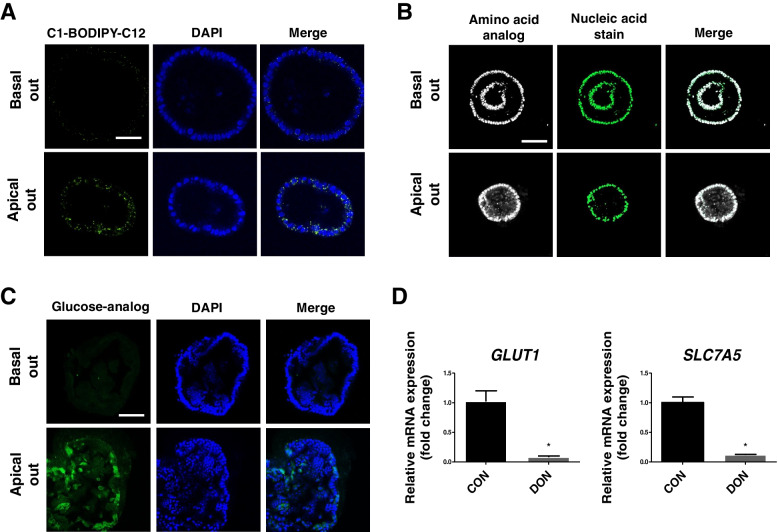
Nutrient absorption ability of chicken intestinal organoid. **A** Fatty acid absorption of the intestinal epithelial layer of the chicken organoid. **B** Amino acid absorption of the intestinal epithelial layer of the chicken organoid. The nuclei were stained with SYTO™ Deep Red Nucleic Acid Stain reagent. **C** Glucose absorption of the intestinal epithelium layer of the chicken organoid. The nuclei were stained with 4′,6-diamidino-2-phenylindole (DAPI; blue). Scale bar = 20 μm. **D** The mRNA expression levels of the transporters when treated with 2 µg/mL deoxynivalenol compared with the untreated group

To assess nutrient uptake in the organoids, we confirmed the nutrient uptake rate using fatty acids, amino acids, and glucose analogs in the organoids. The nutrient uptake rate was examined in the basal-out and apical-out organoids. The fatty acid uptake rate was increased in the apical-out organoids compared to the basal-out organoids (Fig. 5A). The amino acid absorbance and glucose uptake rates were increased in the apical-out organoids compared to the basal-out organoids (Fig. 5B and C). These data suggest that the organoids have similar functionality compared to the actual chicken small intestine and indicate that the apical-out organoid form is required for accurate nutrient absorbance. To confirm the DON-induced disruption of nutrient uptake, basal-out organoids were treated with DON. In the DON-treated group, the mRNA expression levels of glucose transporter-1 (*GLUT-1*) and solute carrier family 7 member 5 (*SLC7A5*) were significantly decreased compared to untreated basal-out organoid cells (Fig. 5D).

## Discussion

DON is produced by *Fusarium* species under the appropriate humidity and temperature conditions and mainly contaminates grains, such as corn, barley, and wheat, in livestock feed [22]. Previous reports have suggested that low doses of DON can induce delayed chicken growth and have a negative effect on chicken health. In addition, DON-contaminated feed may enhance subclinical necrotizing enteritis and the dysfunction of the small intestinal epithelial barrier [23]. Because DON is rapidly absorbed by the small intestinal epithelium, the repeated exposure to DON can result in a high concentration of the mycotoxin. Furthermore, the gut epithelium is comprised of rapidly dividing cells with a high protein turnover rate, which is the primary target of DON [24]. Many reports have demonstrated the DON-induced damage to the reduction of the tight junction proteins via intestinal epithelial cell lines [25–27]. Research regarding DON-induced dysfunction in the small intestine is not complete, and thus, we have developed a stable chicken small intestinal organoid culture system to elucidate DON’s effect on the chicken small intestine.

We confirmed the expression of genetic markers at P1 and P10 using immunocytochemistry to determine the characteristics of the chicken organoids based on the development of the three intestinal sections (duodenum, jejunum, and ileum). As part of its function in nutrient absorption, the small intestinal epithelium contains various cells that are specialized for nutrient transport, including digestive enzymes and hormone secretion, and produces defensins and glycoproteins [28]. Epithelium homeostasis was maintained through the continuous regeneration of its cells from the ISCs that are located in the crypts. *LGR5* is a stem cell molecular marker of self-renewal that regulates the regeneration of the intestinal epithelium [29]. ISCs follow the crypt villus axis and generate the progenitors that differentiate into mature intestinal epithelial cells, including the absorptive enterocyte cells, mucus-producing goblet cells, hormone-secreting enteroendocrine cells, and antimicrobial peptide-secreting Paneth cells [30]. The majority of the intestinal epithelial cells are enterocytes, whose major functions include the absorption and transport of nutrients. [31]. Goblet cells are single-cell glands that produce and secrete mucin. The goblet cells create a mucus layer that acts as a barrier against external pathogens, endotoxins, and microorganisms, and they regulate the intestinal microecological balance and microbial-host immune response [32]. Enteroendocrine cells only represent 1% of all small intestinal epithelial cells, and they detect nutrients and release peptide hormones for digestion; moreover, they can release cytokines when stimulated by pathogens [33]. Tuft cells are found in small numbers within the small intestinal epithelium, accounting for only about 0.4%–2.3% of the total epithelial cell population. They play a role in nutrient uptake, the immune apparatus, and as a physical barrier for the maintenance of homeostasis [34]. In our results, the organoids developed a budded shape over time, which indicates a similarity to the structure of the small intestine. In addition, enterocyte (*Villin*) and enteroendocrine (*CHGA*) markers were confirmed in the organoids, which indicates that the organoids contained various cell types as the ISCs differentiated. No markers were found for Paneth cells (*LYZ*), stem cells (*LGR5*), or goblet cells (*MUC2*). Similarly, no proliferation marker (*KI67*) was detected (Additional file 1). This could be because of the use of improper antibodies in this study.

We confirmed the genetic properties of the chicken organoids in the duodenum, jejunum, and ileum. *MUC2* was significantly increased in the duodenum organoids compared to the actual chicken duodenum, while no differences were observed in the levels of *CHGA*, *CK19*, *LGR5,* and *LYZ*. *DCLK1* was decreased in the duodenum organoids compared to the actual chicken duodenum. The mRNA expression level of *MUC2* was markedly increased in the jejunum organoids compared to the actual chicken jejunum, whereas no differences were observed in the levels of *CHGA, LYZ*, and *CK19*. The mRNA expression levels of *LGR5* and *DCLK* were significantly decreased in the jejunum organoids compared to the actual chicken jejunum. In the ileum, no differences were observed in the levels of *MUC2*, *CHGA*, *CK19*, *LGR5*, *DCLK1,* and *LYZ* compared to the actual chicken ileum. These results may be due to the differences in the budded organoid population. The tuft cells have a very low population rate of about 0.4%–2% in the small intestine and have been reported to appear more numerous when the intestine is damaged [34, 35]. We found that the expression of the tuft cell marker *DLCK1* was decreased compared to the actual chicken small intestines. This may generally be due to a low population ratio, or they may not have been differentiated from the ISCs because of a lack of stimulation during organoid development.

Fatty acids, amino acids, and glucose nutrient analogs were used to confirm the nutrient absorption functions of the organoid. The apical side of the intestinal epithelium is exposed to the lumen, and the associated membrane interacts with environmental stimulation and microorganisms as well as facilitating nutrient absorption [36]. In general, organoid cultures have a basal-out structure with the apical membrane facing forward to the interior, which is different from the organization of the small intestine epithelium and may affect the assessment of the nutrient uptake and barrier function in organoids compared to the actual chicken small intestinal epithelium [37]. Therefore, we developed an apical-out organoid, estimated its permeability, and compared its nutrient intake abilities with those of the basal-out organoids. The 4 kDa FITC-dextran showed that it failed to penetrate both the basal-out and apical-out organoids. In contrast, under long-term incubation of the organoids with FITC-dextran, it could enter the lumen. The 40 kDa FITC-dextran failed to enter either of the organoids. These results may indicate that 4 kDa FITC-dextran can enter into the lumen because of time effect. On the other hand, a few previous results showed that FITC dextran can enter into the lumen within 30 min [17, 38, 39]. These results may be attributed to differences in the culture condition including composition of the medium. Taken together, these results indicate that chicken organoids have a robust intestinal epithelial barrier function, and external antigens of 4 and 40 kDa size could not pass through the intact intestinal barrier. Therefore, we conclude that both organoids mimic the barrier functions of the actual chicken small intestine. These results are limited in their ability to assess whether the correct nutrients are absorbed by the small intestine, since they are transported through the paracellular pathway. Thus, we used fatty acids, amino acids, and glucose analogs to confirm nutrient uptake. We found that the apical-out organoids had a higher rate of nutrient absorbance compared to the basal-out organoids. Some nutrient absorption was also observed in basal-out organoid, these results may have been due to the presence of transporters in the basal membrane. In addition, these experiments were performed after the formation of apical-out organoids to confirm the nutrient uptake ability compared to the basal-out organoids. To construct apical-out organoids, they were treated with EDTA and cultured without Matrigel for 3 d under normal conditions. The apical-out organoid cells showed a high nutrient uptake ability, but these data must be considered regarding the EDTA-mediated intestinal barrier damage, and require additional investigation. Glucose is the main energy source for all animal cells, which is transported across the small intestine epithelium basolateral membrane into the blood circulation by *GLUT-1* [40]. *SLC7A5* is an isoform of the L1 amino acid transporter and serves as a transporter of neutral amino acids, such as leucine, isoleucine, phenylalanine, tryptophan, valine, and methionine [41]. To assess the DON-induced disruption of nutrient uptake ability, the relative mRNA expression levels of the transporters were evaluated. When the organoid cells were treated with DON, the expression levels of the transporters significantly decreased.

The intestinal epithelium plays an important role as a protective barrier against external toxins, pathogens, and allergens from the lumen [42]. Tight junctions are important for the maintenance of the intestinal barrier. They are composed of multiple proteins, including occludin, claudin, and ZO-1, and are located within the apical area between the intestinal cells [43]. Disruption of the intestinal barrier is related to various diseases, including inflammatory bowel disease, celiac disease, type I diabetes, and autism. These disruptions can be influenced by external factors, such as bacterial infections, diseases, food additives, mycotoxins, and other pathogenic infections [44, 45]. We performed a permeability assay and immunostaining of the basal-out and apical-out organoid cells to investigate the relationship between the intestinal barrier function and the DON-induced dysfunction of the intestinal barrier using the chicken organoids. Previous reports have suggested that DON induces defects in the intestinal barrier function by decreasing protein expression in the tight junctions [22, 46]. We confirmed that DON decreased the expression of ZO-1 in the chicken intestinal organoid, which induced chicken intestinal epithelium barrier dysfunction.

## Conclusion

We characterized chicken organoids and confirmed their ability to mimic the nutrient uptake and barrier function of the actual chicken intestinal epithelium. Furthermore, we confirmed that DON-induced intestinal epithelium barrier dysfunction in the organoids. The establishment of a chicken gut organoid culture provides an in vitro model that is genetically and physiologically similar to the actual chicken small intestine in comparison to conventional small intestine epithelial cell lines. This organoid in vitro model can be used to provide an improved understanding of the DON-mediated harmful effects on the diverse small intestine epithelium cells and offers an attractive alternative for future research from an animal welfare perspective.

### Supplementary Information

**Additional file 1: Fig. S1.** Immunostaining of chicken intesinal organoids. **A** Immunofluorescence of MUC2 in chicken intestinal organoid at passage 1. **B** Immunofluorescence of anterial gradient 2 (goblet cell marker) in chicken intestinal organoid at passage 10. **C** Immunofluorescence of LGR5 in chicken intestinal organoid at passage 1 and passage 10. **D** Immunofluorescence of LYZ in chicken intestinal organoid at passage 1 and passage 10. **E** Immunofluorescence of KI67 in chicken intestinal organoid at passage 1 and passage 10. Nuclei were stained with 4', 6-diamidino-2-phenylindole (DAPI; blue). Scale bar = 20 µm. **Fig. S2. **Immunohistochemistry of the chicken small intestine in 20-d embryo. Expression of chromogranin A in chicken small intestine. Nuclei were stained with 4′,6-diamidino-2-phenylindole (DAPI; blue). Scale bar = 20 μm.

## Data Availability

Not applicable.

## Abbreviations

CHGA: Chromagranin A
CK19: Cytokeratin 19
DCLK1: Doublecortin-like kinase 1
DMEM: Dulbecco’s Modified Eagle Medium
DON: Deoxynivalenol
ISCs: Intestinal stem cells
LGR5: Leucine-rich repeat containing G protein-coupled receptor 5
LYZ: Lysozyme
MUC2: Mucin 2
ZO-1: Zonula occludin 1
