# Supplementary material for: Establishment of a chicken intestinal organoid culture system to assess deoxynivalenol-induced damage of the intestinal barrier function
Source: J Anim Sci Biotechnol. 2024 Feb 18;15:30. doi: 10.1186/s40104-023-00976-4 (PMC10874546; doi:10.1186/s40104-023-00976-4)

Supplementary data

Fig. S1. Immunofluorescence of chicken intestinal organoids

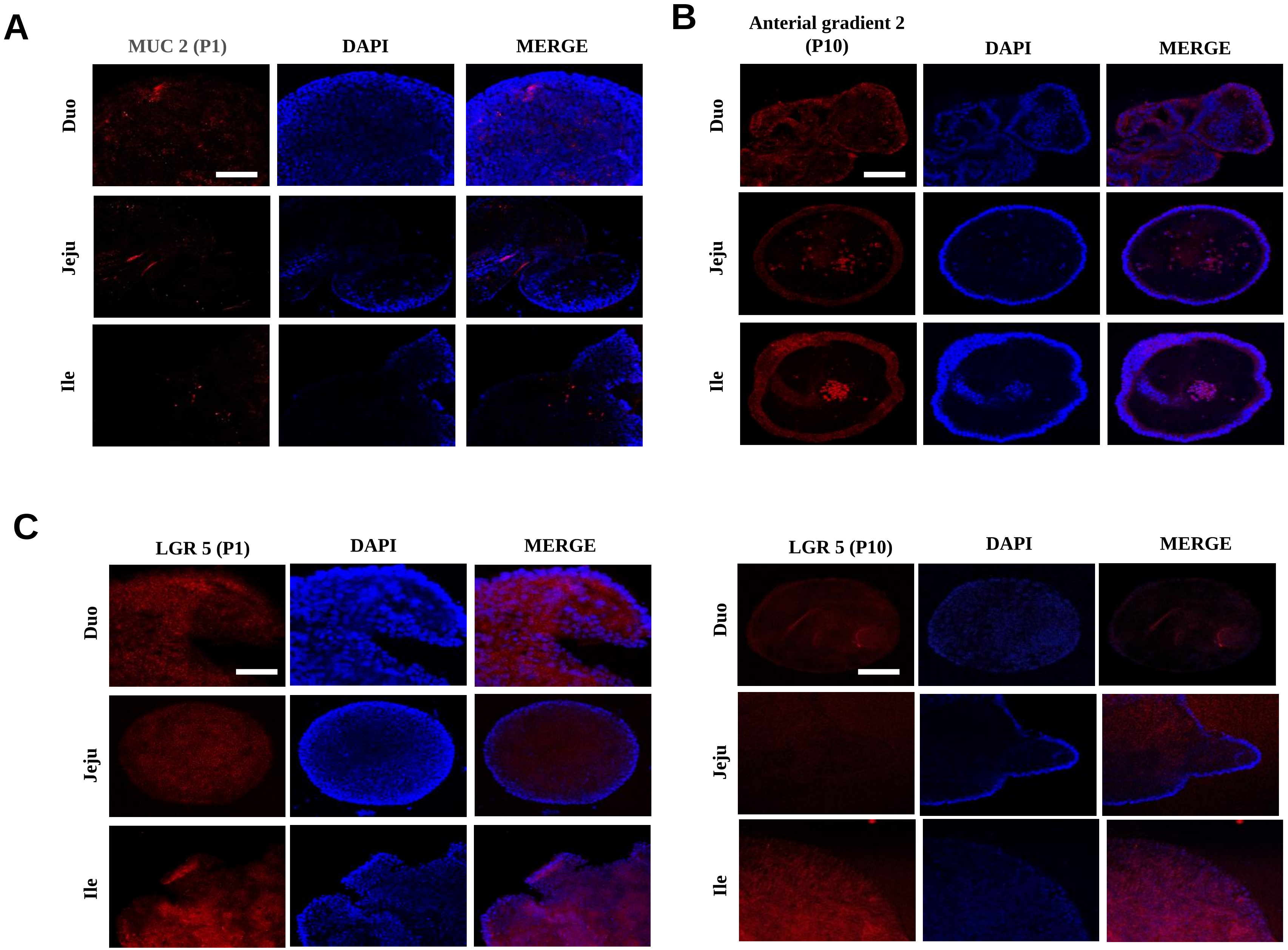

Supplementary data

Fig. S1. Immunofluorescence of chicken intestinal organoids

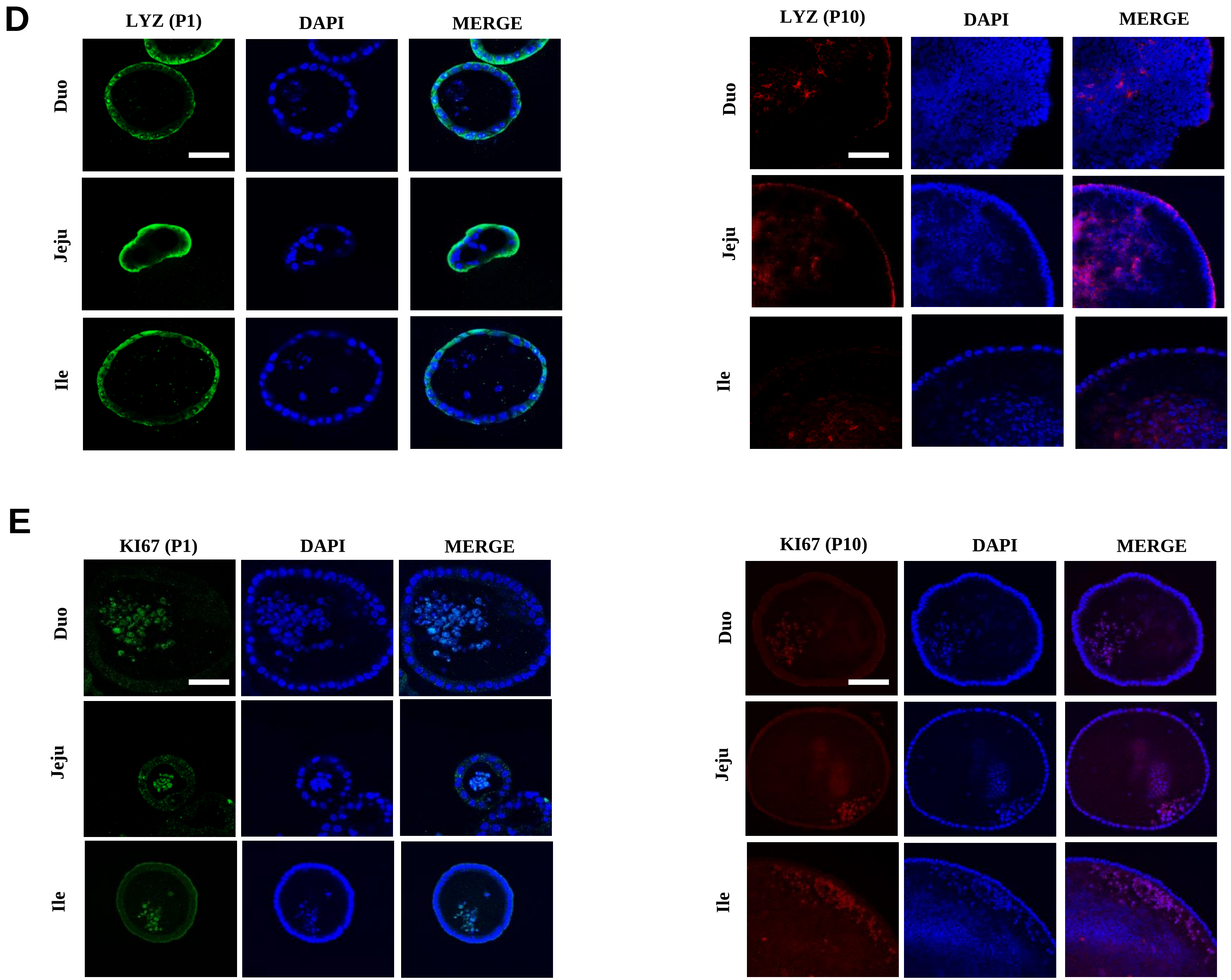

Supplementary data

Fig. S2. Immunohistochemistry of the chicken small intestine in 20-d embryo

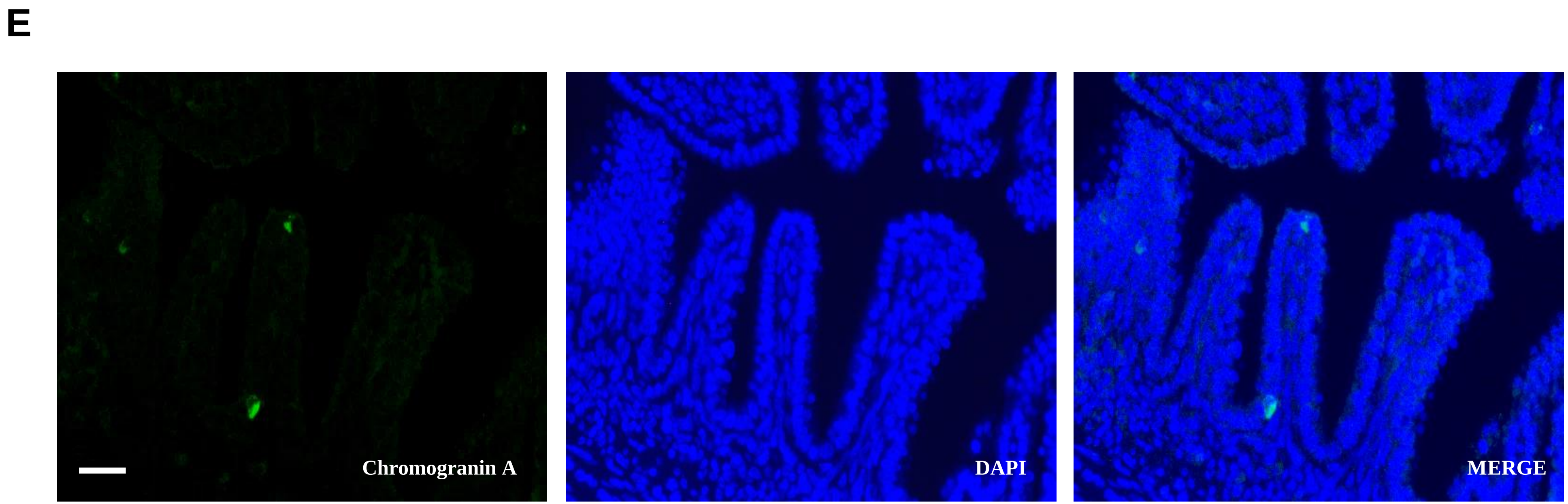

Supplement: Supplementary file 1 — Additional file 1: Fig. S1. Immunostaining of chicken intesinal organoids. A Immunofluorescence of MUC2 in chicken intestinal organoid at passage 1. B Immunofluorescence of anterial gradient 2 (goblet cell marker) in chicken intestinal organoid at passage 10. C Immunofluorescence of LGR5 in chicken intestinal organoid at passage 1 and passage 10. D Immunofluorescence of LYZ in chicken intestinal organoid at passage 1 and passage 10. E Immunofluorescence of KI67 in chicken intestinal organoid at passage 1 and passage 10. Nuclei were stained with 4', 6-diamidino-2-phenylindole (DAPI; blue). Scale bar = 20 µm. Fig. S2. Immunohistochemistry of the chicken small intestine in 20-d embryo. Expression of chromogranin A in chicken small intestine. Nuclei were stained with 4′,6-diamidino-2-phenylindole (DAPI; blue). Scale bar = 20 μm. [file 40104_2023_976_MOESM1_ESM.pdf]
